# Supplementary material for: From sequence to enzyme mechanism using multi-label machine learning
Source: BMC Bioinformatics. 2014 May 19;15:150. doi: 10.1186/1471-2105-15-150 (PMC4229970; doi:10.1186/1471-2105-15-150)
Supplement: Additional file 2 — Java code of ml2db. Additional file ml2db_code.tar.gz contains the Java source code to run the multi-label machine learning experiments and save the results to database. The code’s Javadoc is included. [file 1471-2105-15-150-S2.zip › additional file 2/ml2db/utils/doc/index-files/index-14.html]

N-Index


JavaScript is disabled on your browser.


- Overview
- Package
- Class
- Use
- Tree
- Deprecated
- Index
- Help

- Prev Letter
- Next Letter

- Frames
- No Frames

- All Classes

A B C D E F G H I J K L M N O P Q R S T U V W X Y 


## N

name() - Method in class uk.ac.standrews.utils.main.database.Table


nextDouble() - Method in class cern.jet.random.AbstractDistribution
:   Returns a random number from the distribution.

nextDouble() - Method in class cern.jet.random.engine.RandomEngine
:   Returns a 64 bit uniformly distributed random number in the open unit
    interval `(0.0,1.0)` (excluding 0.0 and 1.0).

nextDouble() - Method in class cern.jet.random.Pareto
:   Returns a random number from the distribution.

nextDouble(double, double) - Method in class cern.jet.random.Pareto
:   Returns a random number from the distribution; bypasses the internal
    state.

nextFloat() - Method in class cern.jet.random.engine.RandomEngine
:   Returns a 32 bit uniformly distributed random number in the open unit
    interval `(0.0f,1.0f)` (excluding 0.0f and 1.0f).

nextInt() - Method in class cern.jet.random.AbstractDistribution
:   Returns a random number from the distribution; returns
    (int) Math.round(nextDouble()).

nextInt() - Method in class cern.jet.random.engine.MersenneTwister
:   Returns a 32 bit uniformly distributed random number in the closed
    interval [Integer.MIN\_VALUE,Integer.MAX\_VALUE] (including
    Integer.MIN\_VALUE and Integer.MAX\_VALUE).

nextInt() - Method in class cern.jet.random.engine.RandomEngine
:   Returns a 32 bit uniformly distributed random number in the closed
    interval [Integer.MIN\_VALUE,Integer.MAX\_VALUE] (including
    Integer.MIN\_VALUE and Integer.MAX\_VALUE);

nextInt() - Method in class uk.ac.standrews.utils.main.stats.TruncatedPareto


nextLong() - Method in class cern.jet.random.engine.RandomEngine
:   Returns a 64 bit uniformly distributed random number in the closed
    interval [Long.MIN\_VALUE,Long.MAX\_VALUE] (including
    Long.MIN\_VALUE and Long.MAX\_VALUE).

nextPseudoInt() - Method in class uk.ac.standrews.utils.main.stats.PseudoTruncatedPareto


nodeHeadToString() - Method in class uk.ac.standrews.utils.main.webutils.simpledomparser.XmlNode
:   String representation of the node opening tag

NONE - Static variable in class uk.ac.standrews.utils.main.diff.Difference


NULL - Static variable in class uk.ac.standrews.utils.main.database.SqlUtils
:   Basic data type: null (the type could not be guessed, eg.

NumberUtils - Class in uk.ac.standrews.utils.main
:   Class

NumberUtils() - Constructor for class uk.ac.standrews.utils.main.NumberUtils


NumberUtilsTest - Class in uk.ac.standrews.utils.test
:   Class

NumberUtilsTest() - Constructor for class uk.ac.standrews.utils.test.NumberUtilsTest

A B C D E F G H I J K L M N O P Q R S T U V W X Y

- Overview
- Package
- Class
- Use
- Tree
- Deprecated
- Index
- Help

- Prev Letter
- Next Letter

- Frames
- No Frames

- All Classes
